# Maternal outcomes of term breech presentation delivery: impact of successful external cephalic version in a nationwide sample of delivery admissions in the United States

**DOI:** 10.1186/s12884-016-0941-9

**Published:** 2016-07-08

**Authors:** Carolyn F. Weiniger, Deirdre J. Lyell, Lawrence C. Tsen, Alexander J. Butwick, BatZion Shachar, William M. Callaghan, Andreea A. Creanga, Brian T. Bateman

**Affiliations:** Department of Anesthesiology and Critical Care Medicine, Hadassah Hebrew University Medical Center, Jerusalem, POB 12000 Israel; Department of Obstetrics and Gynecology, Stanford University School of Medicine, Stanford, CA USA; Department of Anesthesiology, Perioperative and Pain Medicine, Brigham and Women’s Hospital, Harvard Medical School, Boston, MA USA; Department of Anesthesiology, Perioperative, and Pain Medicine, Stanford University School of Medicine, Stanford, CA USA; Maternal-Fetal Medicine Unit, Department of Obstetrics & Gynecology, Sheba Met & March of Dimes Prematurity Research Center, Stanford University School of Medicine, Stanford, CA USA; Division of Reproductive Health, National Center for Chronic Disease Prevention and Health Promotion, Centers for Disease Control and Prevention, Atlanta, GA USA; Department of International Health, International Center for Maternal and Newborn Health, Johns Hopkins Bloomberg School of Public Health, 615 N Wolfe Street, Baltimore, MD USA; Division of Pharmacoepidemiology and Pharmacoeconomics, Department of Medicine, Brigham and Women’s Hospital and Harvard Medical School, Boston, MA USA; Department of Anesthesiology, Critical Care, and Pain Medicine, Massachusetts General Hospital, Harvard Medical School, Boston, MA USA

**Keywords:** Breech, Cesarean delivery, External cephalic version, Maternal morbidity

## Abstract

**Background:**

We aimed to define the frequency and predictors of successful external cephalic version in a nationally-representative cohort of women with breech presentations and to compare maternal outcomes associated with successful external cephalic version versus persistent breech presentation.

**Methods:**

Using the Nationwide Inpatient Sample, a United States healthcare utilization database, we identified delivery admissions between 1998 and 2011 for women who had successful external cephalic version or persistent breech presentation (including unsuccessful or no external cephalic version attempt) at term. Multivariable logistic regression identified patient and hospital-level factors associated with successful external cephalic version. Maternal outcomes were compared between women who had successful external cephalic version versus persistent breech.

**Results:**

Our study cohort comprised 1,079,576 delivery admissions with breech presentation; 56,409 (5.2 %) women underwent successful external cephalic version and 1,023,167 (94.8 %) women had persistent breech presentation at the time of delivery. The rate of cesarean delivery was lower among women who had successful external cephalic version compared to those with persistent breech (20.2 % vs. 94.9 %; *p* < 0.001). Compared to women with persistent breech at the time of delivery, women with successful external cephalic version were also less likely to experience several measures of significant maternal morbidity including endometritis (adjusted Odds Ratio (aOR) = 0.36, 95 % Confidence Interval (CI) 0.24–0.52), sepsis (aOR = 0.35, 95 % CI 0.24–0.51) and length of stay > 7 days (aOR = 0.53, 95 % CI 0.40–0.70), but had a higher risk of chorioamnionitis (aOR = 1.83, 95 % CI 1.54–2.17).

**Conclusions:**

Overall a low proportion of women with breech presentation undergo successful external cephalic version, and it is associated with significant reduction in the frequency of cesarean delivery and a number of measures of maternal morbidity. Increased external cephalic version use may be an important approach to mitigate the high rate of cesarean delivery observed in the United States.

## Background

The last two decades have seen a rise in the frequency of cesarean delivery (CD) in the United States, primarily due to a rise in the rate of primary CD [1]. This trend in CD frequency has been associated with an increase in the rate of maternal morbidity, which is at least partly attributable to the increased risk for adherent placenta in subsequent deliveries, uterine rupture, and maternal hemorrhage [2–4]. Breech presentation accounts for approximately one-fifth of all primary CDs [1, 5].

Cesarean delivery has become the preferred breech delivery option in both the developed and developing world [6, 7] due to improved neonatal outcomes compared with breech vaginal delivery [8]. In the developed world, increased breech CD rates have been reported in the United States, New Zealand, and Europe [1, 9, 10]. In 2010, 16 of 21 European countries reported breech presentation CD rates of greater than 80 %; Norway had the lowest breech presentation CD rate, at 69 % [9].

External cephalic version (ECV) has the potential to reduce the incidence of CD, [11–13] and is endorsed by the American College of Obstetricians and Gynecologists (ACOG) and the Royal College of Obstetricians and Gynaecologists (RCOG) [6, 13]. Successful ECV can facilitate a planned vaginal delivery, which may be associated with reduced maternal morbidity compared with planned CD [14]. Detailed ECV outcomes for women with singleton breech-presentation in the United States have been reported in single-center studies [15, 16]. Maternal outcomes following successful ECV compared with persistent breech presentation delivery have not been examined.

Using a nationally representative database of United States delivery admissions, we aimed to define the frequency and predictors of successful ECV among women with breech presentation in the United States. We also sought to compare maternal outcomes associated with successful ECV versus persistent breech presentation during the delivery admission.

## Methods

### Data source

Data were derived from the Nationwide Inpatient Sample (NIS) (1998–2011) database, maintained by the Agency for Healthcare Research and Quality as part of its Healthcare Cost and Utilization Project, and is the largest all-payer discharge database in the United States. Additional details regarding the database are available at: http://www.hcup-us.ahrq.gov/.

The NIS contains demographic data such as age, race, and the primary expected payer for the hospital admission. Diagnoses and procedures associated with each admission are coded using the International Classification of Diseases, Ninth Revision, Clinical Modification manual (ICD-9-CM). Each year approximately 1000 hospitals, representing all types of health facilities, are selected for the NIS database. Hospitals are selected for inclusion in order to create a sample that is maximally representative of all types of health facilities in United States admissions. All admissions data from these hospitals are included in the database; this dataset includes information on approximately 20 % of United States hospital admissions. Using analytical sample weights can provide national estimates derived using the data.

### Cohort

We identified women admitted for delivery who had a breech presentation or a successful ECV during pregnancy using ICD-9-CM codes, using the NIS database during 1998–2011, as has been utilized previously [17, 18]. A code for successful ECV, 652.1x, identified women whose fetus had previously been breech and had undergone successful version to cephalic presentation (either before or during the delivery admission). The breech presentation group, 652.2x, comprised all women with persistent breech presentation during the delivery admission; there were no codes that can differentiate women who had an unsuccessful ECV attempt from those who did not undergo ECV prior to the delivery admission.

Based on the most recent ACOG and RCOG guidelines for ECV, and recommendations from clinical experts, [6, 13, 19] we excluded: admissions with spontaneous preterm labor and delivery (below 37 complete weeks); conditions that are relative or absolute contraindications to performing ECV including multiple gestations, prior CD, human immunodeficiency virus (HIV), placenta previa or accreta, uterine anomalies, oligohydramnios; and maternal age <16 years. The exclusion criteria were identified by ICD-9-CM diagnosis codes recorded at the time of admission for delivery.

### Analysis

We examined patient- and hospital-level factors associated with successful ECV. Specific patient, obstetric and hospital characteristics were selected *a priori* as potential predictors of successful ECV, based on our literature review and clinical plausibility [6, 13, 20]. For patient characteristics, we assessed: maternal age (categorized as 16 to 19 years, 20 to 34 years, 35 to 39 years, and 40 years or older), race/ethnicity (categorized as white, black, Hispanic, Asian/Pacific Islander, other, and missing), and primary expected payer (categorized as Medicaid, Medicare, Private Insurance, self-pay, no charge, and other). For obstetric factors, we assessed: diabetes (gestational and pre-existing), hypertensive disease of pregnancy, grandmultiparity, and fetal growth restriction. Individual hospital characteristics were assessed, including: annual hospital delivery volume (categorized as < =1,000, 1,001 to 2,000, 2,001 to 4,000, and >4,000 deliveries per year) and annual hospital CD frequency (categorized as <20 %, 20–24 %, 25–29 %, 30–34 %, ≥35 %). All covariates were entered into a multivariable logistic regression model to examine predictors of successful ECV (versus persistent breech). Adjusted odds ratios (aOR) and 95 % confidence intervals (CI) are reported.

To compare maternal outcomes between women who underwent successful ECV versus those with persistent breech presentation at delivery, we fit separate multivariable logistic regression models for the following conditions: CD, thromoboembolic phenomena, anesthesia complications, chorioamnionitis, endometritis, sepsis, blood transfusion, hysterectomy, and length of stay greater than 7 days. We also performed linear regression analyses to assess independent associations between successful ECV versus persistent breech presentation at the delivery admission and total hospital length of stay and hospital charges. All regression models were adjusted for the demographic, obstetrical, and hospital characteristics noted above.

Information on attempted ECV during the delivery admission was available in our database using the ICD-9-CM procedure code 73.91. This enabled a secondary analysis of maternal outcomes for *attempted* ECV performed during the delivery admission.

This secondary analysis was restricted to ECV performed during the delivery admission, ICD-9-CM procedure code 73.91 (during delivery admission ECV would only be performed for breech presentation) versus without ECV attempt (all other women with persistent breech presentation at delivery, 652.2x). Successful ECV during the delivery admission was available in our database using the ICD-9-CM code 652.1x for women who had attempted ECV during the delivery admission (ICD-9-CM procedure code 73.91).

All analyses accounted for the complex survey design of the NIS [4, 17]. Statistical analyses were performed using Stata Statistical Software: Release 12.1 (StataCorp LP, College Station, TX). Admissions with missing payer (*n* = 2481) or age (*n* = 690) were excluded from adjusted analyses.

## Results

Between 1998 and 2011, there were 1,861,574 delivery admissions with successful ECV or persistent breech presentation. Of these, 781,998 (42 %) women underwent pre-term delivery or were coded in the NIS database with at least one relative or absolute contraindication to ECV and were excluded from the cohort. Our leading reasons for exclusion from the cohort were spontaneous preterm delivery (*n* = 388,863), prior CD (*n* = 287,728), multiple gestations (*n* = 141, 758), and oligohydramnios (*n* = 120,962). The full exclusions are presented in Table 1. In the final analytic cohort, 56,409 (5.2 %) women underwent successful ECV during or prior to the delivery admission and 1,023,167 (94.8 %) women had persistent breech presentation at the time of delivery.

**Table 1:** Exclusion from the cohort based for relative or absolute contraindications to external cephalic version

Exclusion criteria	Number of women excluded = 718, 998*
Spontaneous preterm labor	388, 863
Prior cesarean delivery	287, 728
Multiple gestations	141, 758
Oligohydramnios	120, 962
Placenta previa	23,207
Uterine anomaly	17, 248
Age <16 years	7457
Potential placenta accreta	1542
Human immunodeficiency virus	551

*These reasons are not mutually exclusive and any woman could be excluded for more than one relative contraindication

Maternal demographic, clinical, and hospital characteristics are presented in Table 2. Compared to women aged between 20 to 34 years, younger women were less likely and older women more likely to have undergone successful ECV. Compared to women whose deliveries were covered by Medicaid (low- to no-income families and individuals), those covered by Medicare or private insurance were less likely to have a successful ECV. Women who underwent successful ECV were less likely to have hypertensive disease or a fetus with intrauterine growth restriction (IUGR) and more likely to be grandmultiparas (≥5 previous deliveries). There was a strong inverse relationship between annual hospital CD frequency and successful ECV. Successful ECV was approximately one-third less likely among women who delivered at hospitals with annual CD frequency ≥35 %, compared to women who delivered at hospitals with low annual CD frequency, <20 %, controlling for other factors (Table 2). No association was observed between annual hospital delivery volume and frequency of successful ECV.

**Table 2:** Maternal demographic, clinical and hospital characteristics and their association with successful external cephalic version among breech presentations (*N* = 1, 079, 576)

	Successful ECV (*N* = 56, 409) *N* (%)	Persistent breech presentation at Delivery (*N* = 1, 023, 167) *N* (%)	Adjusted Odds Ratio (95 % CI)
Age (years):			
16–19	3355 (5.9 %)	79, 603 (7.8 %)	0.73 (0.64–0.83)
20–34	41, 551 (73.7 %)	765, 573 (74.4 %)	Reference
35–39	9102 (16.1 %)	143, 789 (14.1 %	1.21 (1.13–1.3)
> = 40	2395 (4.2 %)	37, 517 (3.7 %)	1.25 (1.12–1.39)
Race/Ethnicity:			
White	25, 795 (45.7 %)	490, 605 (47.9 %)	Reference
Black	2936 (5.2 %)	62, 567 (6.1 %)	0.93 (0.83–1.04)
Hispanic	6797 (12.0 %)	145, 623 (14.2 %)	0.90 (0.81–1.00)
Asian	1711 (3.0 %)	40, 104 (3.9 %)	0.8 (0.69–0.91)
Pacific Islander	1987 (3.5 %)	39, 884 (3.9 %)	0.95 (0.83–1.09)
Other/ Unknown	17, 182 (30.5 %)	244, 384 (23.9 %)	1.24 (0.96–1.60)
Primary expected payer:			
Medicare	200 (0.4 %)	5242 (0.5 %)	0.68 (0.48–0.95)
Medicaid	17, 520 (31.1 %)	322, 282 (31.6 %)	Reference
Private insurance	35, 157 (62.5 %)	636, 748 (62.4 %)	0.92 (0.87–0.99)
Self-Pay	1734 (3.1 %)	29, 823 (2.9 %)	1.00 (0.87–1.14)
No charge	85 (0.2 %)	2078 (0.2 %)	0.76 (0.44–1.30)
Other	1557 (2.8 %)	24, 670 (2.4 %)	1.09 (0.95–1.24)
Hypertensive disease	3197 (5.7 %)	75, 464 (7.4 %)	0.78 (0.72–0.86)
Diabetes	3375 (6.0 %)	64, 546 (6.3 %)	0.97 (0.9–1.05)
Intrauterine growth restriction	777 (1.4 %)	21, 853 (2.1 %)	0.69 (0.58–0.82)
Grand-multiparity	862 (1.5 %)	8401 (0.8 %)	1.81 (1.50–2.18)
Annual hospital delivery volume:			
<=1000	11, 286 (20.0 %)	201, 914 (19.7 %)	Reference
1001–2000	14, 118 (25.0 %)	236, 610 (23.1 %)	1.09 (0.93–1.28)
2001–4000	18, 063 (32.0 %	357, 615 (35.0 %)	0.97 (0.84–1.12)
>4000	12, 942 (22.9 %)	227, 028 (22.2 %)	1.18 (0.84–1.65)
Annual hospital cesarean delivery frequency (%):			
<20	9698 (17.2 %)	103, 493 (10.1 %)	Reference
20–24	13, 686 (24.3 %)	194, 484 (19.0 %)	0.75 (0.62–0.90)
25–29	16, 345 (29.0 %)	292, 255 (28.6 %)	0.60 (0.49–0.73)
30–34	9736 (17.3 %)	217, 125 (21.2 %)	0.48 (0.37–0.64)
> = 35	6943 (12.3 %)	215, 810 (21.1 %)	0.35 (0.29–0.43)

ECV = External Cephalic Version; IUGR = Intrauterine growth retardation

Adjusted for all characteristics in the table; All percentages weighted. Note age is missing for 690 patients and primary payer for 2481 patients. The odds presented are the odds of having successful external cephalic version versus persistent breech delivery

Among women who underwent successful ECV, the CD frequency was 20.2 % whereas the CD frequency among women with persistent breech presentation delivery was 94.9 % (*P* < 0.001). Table 3 shows the association between successful ECV and maternal obstetrical outcomes, adjusted for potentially confounding variables. Compared to women with persistent breech presentation, women who underwent successful ECV had a lower odds of developing endometritis (aOR 0.36, 95 % CI 0.24–0.52), sepsis (aOR 0.35, 95 % CI 0.24–0.51), length of stay > 7 days (aOR 0.53, 95 % CI 0.40–0.70), but a higher odds of chorioamnionitis (aOR 1.83, 95 % CI 1.54–2.17). Hospital charges were lower (-$1,122 (95 % CI -$1,464 to–$781)), and likewise, the duration of admission was reduced (-0.47 days (95 % CI -0.52 to–0.42)) for women with successful ECV versus women with persistent breech presentation at the delivery admission, adjusted for other patient and hospital characteristics.

**Table 3:** Maternal outcomes following successful external cephalic version compared with breech presentation delivery

	Successful ECV (*N* = 56, 409) *N* (%)	Persistent Breech Presentation at Delivery *N* = (1, 023, 167) *N* (%)	Adjusted Odds Ratio (95 % CI)
Cesarean delivery	11, 381 (20.2 %)	970961 (94.9 %)	0.013 (0.011–0.014)
Thromboembolic phenomena	36 (0.1 %)	1024 (0.1 %)	0.64 (0.30–1.38)
Anesthesia complications	**	379 (0.04 %)	0.47 (0.12–1.73)
Chorioamnionitis	924 (1.6 %)	8884 (0.9 %)	1.83 (1.54 to 2.17)
Endometritis	132 (0.2 %)	6518 (0.6 %)	0.36 (0.24–0.52)
Sepsis	132 (0.3 %)	6638 (0.7 %)	0.35 (0.24–0.51)
Blood Transfusion	301 (0.5 %)	7325 (0.7 %)	0.85 (0.65–1.11)
Hysterectomy	42 (0.1 %)	821 (0.1 %)	0.89 (0.44–1.80)
Length of stay > 7 days	328 (0.6 %)	12, 604 (1.2 %)	0.53 (0.40–0.70)

ECV = External cephalic version; **Cell sizes less than 11 cannot be disclosed in accordance with data use agreement; Adjusted Odds Ratio (95 % CI); Adjusted for all characteristics in Table 1; All percentages weighted. The codes for endometritis are included in the definition for maternal sepsis. The odds presented are the odds of having the maternal outcome associated with successful external cephalic version compared with breech presentation delivery

In the secondary analysis of attempted ECV performed *during* the delivery admission versus without ECV attempt, ECV was attempted in 26,455 (2.5 %) women with breech presentation at delivery admission, with an ECV success rate (ICD-9-CM code, 652.1x) of 64.8 %. The associations between attempted ECV during the delivery admission and maternal morbidities are presented in Table 4. The CD rate was significantly lower among women who attempted ECV during the delivery admission, versus without ECV attempt (39.2 % vs. 95.1 % respectively, aOR 0.03 (95 % CI 0.03–0.04)). Among women who attempted ECV during the delivery admission versus without ECV attempt, the odds of a hospital length of stay > 7 days were significantly lower (aOR = 0.57; 95 % CI 0.39–0.83).

**Table 4:** Maternal outcomes following attempted external cephalic version at delivery admission among women with persistent breech presentation

	ECV attempted (*N* = 26,455) *N* (%)	No ECV attempted (*N* = 1,013,855) *N* (%)	Adjusted Odds Ratio (95 % CI)
Cesarean delivery	10, 357 (39.2 %)	963, 706 (95.1 %)	0.03 (0.03–0.04)
Thromboembolic phenomena	26 (0.1 %)	1024 (0.1 %)	0.97 (0.40–2.36)
Anesthesia complications	**	374 (0.0 %)	0.49 (0.07–3.49)
Chorioamnionitis	268 (1.0 %)	8807 (0.9 %)	1.14 (0.87 to 1.49)
Endometritis	130 (0.5 %)	6442 (0.6 %)	0.77 (0.52–1.13)
Sepsis	130 (0.5 %)	6561 (0.6 %)	0.76 (0.51–1.11)
Blood transfusion	121 (0.5 %)	7281 (0.7 %)	0.75 (0.47–1.19)
Hysterectomy	19 (0.1 %)	812 (0.1 %)	0.81 (0.30–2.19)
Length of stay > 7 days	167 (0.6 %)	12, 536 (1.2 %)	0.57 (0.39–0.83)

ECV = External cephalic version; **Cell sizes less than 11 cannot be disclosed in accordance with data use agreement; The codes for endometritis are included in the definition for maternal sepsis. Adjusted Odds Ratio (95 % CI); Adjusted for all characteristics in Table 1; All percentages weighted

## Discussion

Using a nationwide sample of delivery admissions at term gestation, we observed that only 1 in 20 women with breech presentation had undergone a successful ECV. Successful ECV was associated with a reduction in certain measures of maternal morbidity and healthcare utilization compared with persistent breech presentation at delivery. The strong inverse relationship between successful ECV and the annual hospital CD frequency suggests that ECV performance may vary according to institutional delivery practices [21]. Our finding extends a recent observation by Rosenstein et al. that the frequency of attempted vaginal birth after cesarean delivery is inversely correlated with the primary CD frequency among nulliparous women with low-risk vertex singleton term pregnancies [22]. This suggests there may be provider-level, patient-level, and hospital-level practices that impact use of measures to avoid cesarean delivery, and that ECV is one such practice.

Management of persistent breech by means of ECV represents a paradigm shift from breech CD or vaginal breech delivery [23]. However, based on the high frequency of breech CD in the United States population, [1] ECV has not been widely performed. In two single center studies in the United States and United Kingdom, ECV was performed on less than half the women with breech presentation, [15, 24] and in 11 African countries, only 20 % of practitioners offered ECV [25]. Despite the previously established connection between successful ECV, vaginal delivery and improved maternal outcomes, we report that ECV was infrequently performed.

Hesitancy to offer or use ECV is often related to parental or provider concerns for maternal discomfort or potential complications, including the 1-2 % incidence of placental abruption and preterm rupture of the membranes (PROM) [26–28]. The low frequency of ECV use may stem from limited provider experience, or from discomfort in counseling on breech management options, [29] or performing the technique. Clinicians will need to overcome barriers for more widespread use of ECV to occur.

Women who underwent successful ECV had a reduced likelihood of several measures of maternal morbidity compared to women with persistent breech presentations. This finding likely relates to the high CD frequency observed among women who had persistent breech presentation; Liu et al., reported significant maternal morbidities such as cardiac arrest, hemorrhage, hematoma, hysterectomy, infection, thromboembolic phenomena and anesthesia complications associated with planned cesarean versus vaginal delivery [14]. Moreover, women with successful ECV in our study had lower measures of healthcare utilization including hospital costs and length of stay; this finding is consistent with cost analyses for successful ECV that indicates a reduction in hospital costs through increased vaginal delivery practice [30].

Our study is subject to several limitations. First, the NIS database lacks a specific code for unsuccessful ECV prior to admission for delivery. As a consequence, our persistent breech presentation cohort contains a combination of women with no ECV attempt as well as those with a failed ECV attempt. Furthermore, because we restricted our analysis to delivery admissions coded as successful ECV (652.1x) or breech presentation at delivery (652.2x), it is also possible that some women had successful ECV performed prior to the delivery admission, and then underwent vaginal delivery (without 652.2 or 652.1). However, our overall frequency of observed breech presentations was consistent with other sources, suggesting that this kind of miscoding is uncommon or balanced, and that the diagnostic codes likely captured most of the breech presentation [31].

Second, maternal and infant admissions cannot be linked with the NIS database, such that we are unable to report perinatal and neonatal morbidities associated with ECV and breech presentation [8, 32]. Future studies should more closely examine the frequency and relationship between maternal and fetal/neonatal risks associated with ECV attempts. An analysis of the 2006 national U.S. data reported that neonatal outcomes for deliveries following successful ECV were similar to non-ECV breech presentation; however, a failed attempted ECV was associated with increases in abnormal fetal heart rate tracings, need for assisted ventilation, and neonatal intensive care admission [32]. Third, the NIS lacks clinically nuanced data relevant to an ECV attempt such as placental location or breech type, as well potentially important elements (e.g., parity, prior vaginal birth after cesarean delivery, body mass index, or gestational age at delivery) that could affect or contraindicate ECV; we excluded 42 % of delivery admissions due to potential and relative contraindications to ECV [6, 13, 19]. In contrast to other smaller databases that reported exclusion criteria of less than 32 % of the population, [16, 33] our conservative approach excluded women who some clinicians might consider as appropriate candidates for ECV, such as women with oligohydramnios and a history of prior CD [34, 35].

## Conclusion

In conclusion, a low proportion of women with breech presentation undergo successful ECV in the United States. Breech presentation has a disproportionately high impact on the increasing rate of CD, relative to its frequency in the term pregnant population. The frequencies of successful ECV and annual hospital CDs are inversely correlated. Furthermore, ECV is associated with a significant reduction in the frequency of CD and a number of measures of maternal morbidity. These findings indicate that increasing the frequency of ECV may be an important approach to mitigate the high rate of CD observed in the United States.

## Abbreviations

ACOG, American College of Obstetrics and Gynecology; aOR, adjusted Odds Ratio; CD, cesarean delivery; CI, Confidence Interval; ECV, external cephalic version; HIV, human immunodeficiency virus; ICD-9-CM, International Classification of Diseases, Ninth Revision, Clinical Modification; IUGR, intrauterine growth retardation; NIS, Nationwide Inpatient Sample; PROM, preterm rupture of the membranes; RCOG, Royal College of Obstetrics and Gynaecology
